# Intravascular ultrasound-guided versus angiography-guided percutaneous coronary intervention for acute myocardial infarction with cardiogenic shock

**DOI:** 10.1038/s41598-024-59723-y

**Published:** 2024-05-01

**Authors:** Oh-Hyun Lee, Seok-Jae Heo, Thomas W. Johnson, Yongcheol Kim, Deok-Kyu Cho, Jung-Sun Kim, Byeong-Keuk Kim, Donghoon Choi, Myeong-Ki Hong, Yangsoo Jang, Myung Ho Jeong, The Investigators for KAMIR, Myung Ho Jeong, Tae Hoon Ahn, Ki Bae Seung, Seung-Woon Rha, Hyo-Soo Kim, Chang-Hwan Yoon, Hyeon-Cheol Gwon, Chong-Jin Kim, Junghan Yoon, In-Whan Seong, Kyung-Kuk Hwang, Jei Keon Chae, Seok Kyu Oh, Jung-Hee Lee, Shung Chull Chae, Seung-Ho Hur, Kwang Soo Cha, Jin-Yong Hwang, Doo-Il Kim, Seung-Jae Joo, Myung Ho Jeong, KiYuk Chang, Hee-Yeol Kim, Ki-Dong Yoo, Sang-Yong Yoo, Shung Chull Chae, Jin-Yong Hwang, Weon Kim, Seung Ho Hur, Seung-Woon Rha, Deuk-Young Nah, Chong-Jin Kim, Kwang Soo Cha, Chang-Hwan Yoon, Hyo-Soo Kim, Hyeon-Cheol Gwon, Jang Hyun Cho, Jong-Seon Park, Seok Kyu Oh, Junghan Yoon, Kyu-Sun Lee, Jei Keon Chae, Jay-Young Rhew, Seung-Jae Joo, Yong-Mo Yang, In-Whan Seong, Kyung-Kuk Hwang, Sang-Hyun Kim, Doo-Il Kim, Yong Hwan Park, Sung Uk Kwon, Tae Hoon Ahn, Dong-Bin Kim, Byung Ryul Cho, Seung-Uk Lee, Jang Ho Bae, Sang Yup Lim, Kee Sik Kim, Moo Hyun Kim, Sang-Ho Park, Seung Je Tak, Sung-Il Woo, Byung Ok Kim

**Affiliations:** 1 Yonsei University College of Medicine and Cardiovascular Center, Yongin Severance Hospital, 363 Dongbaekjukjeon-Daero, Giheung-Gu, Yongin, 16995 Republic of Korea; 2 Division of Biostatistics, Department of Biomedical Systems Informatics, Yonsei University College of Medicine, Seoul, Republic of Korea; 3 Bristol Heart Institute, Bristol, UK; 4 Severance Cardiovascular Hospital, Yonsei University Health System, Seoul, Republic of Korea; 5 Department of Cardiology, CHA Bundang Medical Center, CHA University School of Medicine, Seongnam, Republic of Korea; 6 Chonnam National University Hospital and Medical School, Gwangju, Republic of Korea; 7 Department of Cardiology, Principal investigator of the Korea Acute Myocardial Infarction Registry, Chonnam National University Hospital, 42 Jebong-Ro, Dong-Gu, Gwangju, 61469 Republic of Korea; 8 Chonnam National University Hospital, Gwangju, South Korea; 9 Gachon University Gil Medical Center, Incheon, South Korea; 10 Seoul St. Mary’s Hospital, Seoul, South Korea; 11 Korea University Guro Hospital, Seoul, South Korea; 12 Seoul National University Hospital, Seoul, South Korea; 13 Seoul National University Bundang Hospital, Seongnam-si, South Korea; 14 Samsung Medical Center, Seoul, South Korea; 15 Gangdong Kyunghee University Hospital, Seoul, South Korea; 16 Wonju Severance Christian Hospital, Wonju, South Korea; 17 Chungnam National University Hospital, Daejeon, South Korea; 18 Chungbuk National University Hospital, Cheongju-si, South Korea; 19 Chonbuk National University Hospital, Jeonju-si, South Korea; 20 Wonkwang University Hospital, Iksan-si, South Korea; 21 Yeungnam University Medical Center, Daegu, South Korea; 22 Kyungpook National University Hospital, Daegu, South Korea; 23 Keimyung University Dongsan Medical Center, Daegu, South Korea; 24 Pusan Natinoal University Hospital, Busan, South Korea; 25 Gyeongsang National University Hospital, Jinju-si, South Korea; 26 Inje University Haeundae Paik Hospital, Busan, South Korea; 27 Jeju National University Hospital, Jeju-si, South Korea; 28 Bucheon St. Mary’s Hospital, Bucheon-si, South Korea; 29 St. Vincent Hospital, Darlinghurst, Australia; 30 Gangneung Asan Hospital, Gangneung-si, South Korea; 31 Kyunghee University Hospital, Seoul, South Korea; 32 Dongguk University Gyeongju Hospital, Gyeongju-si, South Korea; 33 Pusan National University Hospital, Busan, South Korea; 34 Suncheon St. Garolo Hospital, Suncheon-si, South Korea; 35 Yeungnam University Hospital, Daegu, South Korea; 36 Daejeon Eulji University Hospital, Daejeon, South Korea; 37 Prebyterian Medical Center, New York, USA; 38 Cheongju St. Mary’s Hospital, Cheongju-si, South Korea; 39 Seoul Boramae Hospital, Seoul, South Korea; 40 Samsung Changwon Hospital, Changwon-si, South Korea; 41 Inje University Ilsan Paik Hospital, Goyang-si, South Korea; 42 St. Paul’s Hospital, Vancouver, Canada; 43 Kangwon National University Hospital, Chuncheon-si, South Korea; 44 Kwangju Christian Hospital, Gwangju, South Korea; 45 Konyang University Hospital, Daejeon, South Korea; 46 Korea University Ansan Hospital, Ansan-si, South Korea; 47 Daegu Catholic University Medical Center, Daegu, South Korea; 48 Dong-A University Hospital, Busan, South Korea; 49 Soonchunhyang University Cheonan Hospital, Cheonan-si, South Korea; 50 Ajou University Hospital, Suwon-si, South Korea; 51 Inha University Hospital, Incheon, South Korea; 52 Inje University Sanggye Paik Hospital, Seoul, South Korea

**Keywords:** Cardiology, Interventional cardiology

## Abstract

The benefits of intravascular ultrasonography (IVUS)-guided percutaneous coronary intervention (PCI) in the clinical context of cardiogenic shock (CS) complicating acute myocardial infarction are lacking. We aimed to investigate the impact of IVUS-guided PCI in patients with AMI and CS. From the pooled data based on a series of Korean AMI registries during 2011–2020, we identified 1418 consecutive patients who underwent PCI with second generation drug-eluting stent (DES) for AMI and CS. The primary endpoint was the 1-year rate of target lesion failure (TLF), defined as the composite of cardiac death, target vessel myocardial infarction, and ischemic-driven target lesion revascularization. In total, 294 (20.7%) and 1124 (79.3%) underwent IVUS-guided and angiography-guided PCI with second generation DES implantation, respectively. The 1-year TLF was not significantly different between groups after IPTW analysis (hazard ratio 0.93, 95% confidence interval 0.65–1.34, p = 0.70). Additionally, the adjusted landmark analysis for TLF at 30 days and between 30 days and 1 year after PCI demonstrated no significant difference between the groups. In conclusion, in patients with AMI and CS who underwent PCI with second-generation DES, IVUS-guided PCI did not improve the 1-year TLF compared with angiography-guided PCI.

**Registration: ** URL: http://cris.nih.go.kr. KCT0000863 and KCT0008355.

## Introduction

Cardiogenic shock (CS) accompanies 3–10% of acute myocardial infarctions (AMIs), with combined AMI and CS showing worse short- and long-term clinical outcomes than AMI without CS^1^ Despite mortality reduction by 40–50% from early revascularization^2^, AMI complicated by CS remains a leading cause of death^3^. This is further evidenced by a recent increase in mortality rates owing to an increase in comorbidities in the aging population^4^. Therefore, an early invasive strategy with appropriate revascularization is crucial for successful treatment in this population^2^, along with critical care including fluid administration and inotropic supports^5^.

Intravascular ultrasonography (IVUS) offers critical insights on lesion characteristics, enabling optimal stent deployment. IVUS also plays a pivotal role in detecting suboptimal stent results and improving percutaneous coronary intervention (PCI) outcomes^6^. Therefore, it has been widely used in the contemporary PCI era^7^. Two recent AMI registries demonstrated that IVUS-guidance improved the long-term clinical outcomes of PCI^8,9^.

However, data regarding the benefits of IVUS-guided PCI in the clinical context of CS complicating AMI are lacking. Therefore, this study aimed to investigate the clinical impact of IVUS-guided versus angiography-guided PCI in patients with AMI and CS.

## Methods

### Patients

The Korean Acute Myocardial Infarction Registry (KAMIR) registry was designated to evaluate real-world long-term clinical outcomes of patients with AMI. The KAMIR-National Institute of Health (NIH) (KCT-0000863) includes patients with AMI between November 2011 and December 2015^10^, whereas the KAMIR-V (KCT-0008355) includes patients with AMI between January 2016 and June 2020^11^. These registries encompass nationwide, multicenter and prospective observational cohorts supported by the Korean Working Group of Acute Myocardial Infarction. The 20 and 43 centers that participated in the KAMIR-NIH and KAMIR-V, respectively, were equipped for primary PCI and on-site cardiac surgery. The ethics committees of each participating center approved the study protocol. This study complied with the tenets of the Declaration of Helsinki. All patients provided written informed consent upon enrollment.

**Figure 1:**
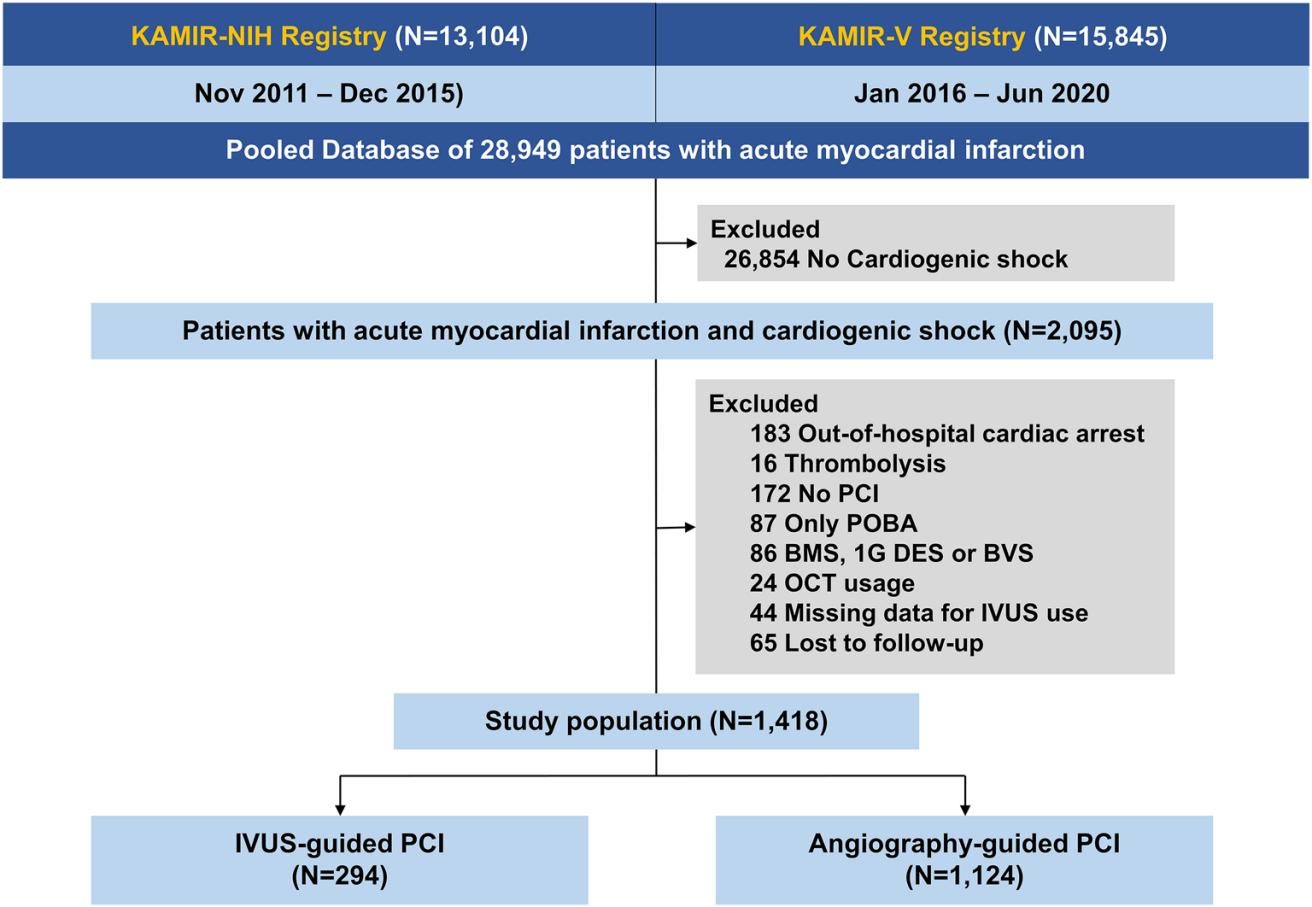
Study flowchart.

Among 28,949 consecutive patients with AMI between 2011 and 2020, we selected 2,095 patients with AMI and CS (Fig. 1). CS was defined as systolic blood pressure < 90 mmHg for > 30 min or the need for supportive management to maintain systolic blood pressure > 90 mmHg; clinical signs of pulmonary congestion; and evidence of impaired end-organ perfusion with at least one of the following: cool extremities, decreased urine output, increased lactic acid level, or altered mental status^2^. The exclusion criteria were no CS; out-of-hospital cardiac arrest; thrombolysis; no PCI or history of PCI without stenting; treatment with a bare metal stent, first-generation DES, or bioresorbable vascular scaffold; history of optical coherence tomography guidance; missing data for IVUS use; and loss to follow-up. Patients who were discharged but never visited the outpatient department again were considered lost to follow-up.

### Treatment

Patients diagnosed with AMI were treated according to contemporary guidelines^12,13^. The strategy for revascularization, techniques, vascular access, selection of devices, and adjunctive antithrombotic therapy including glycoprotein IIb/IIIa inhibitor were left to the discretion of each physician. All procedures were performed in accordance with standard interventional techniques. The decision to use IVUS was at the operator’s discretion. Patients were recommended to receive optimal pharmacological therapy after PCI according to standard guidelines. For cardiogenic shock management, the use of intra-aortic balloon pump (IABP), extracorporeal membrane oxygenation (ECMO), and inotropic agents was also determined at the operator’s discretion.

### Endpoints

The primary endpoint was target lesion failure (TLF), defined as the composite of cardiac death, target vessel myocardial infarction (TV-MI), and ischemia-driven target lesion revascularization (ID-TLR) at 1 year after the index procedure. The secondary endpoints included all-cause mortality; individual components of TLF; definite/probable stent thrombosis as defined by the Academic Research Consortium^14^; and major adverse cardiovascular events (MACEs), including death from any cause, myocardial infarction (MI), and revascularization. Clinical outcomes at 30 days and between 30 days and 1 year were also compared. All-cause mortality was regarded as cardiac death unless a definite noncardiac cause was identified. TV-MI was defined as MI with evidence of myocardial necrosis in the vascular territory of a previously treated target vessel. Target lesion revascularization was considered ischemia-driven if any revascularization including PCI or bypass surgery for the target lesion was performed in ≥ 50% angiographic diameter stenosis with ischemic symptoms, positive results on a functional study, or ≥ 70% angiographic diameter stenosis with or without documented ischemia.

### Statistical analysis

Data are presented as the mean ± standard deviation (SD) for continuous variables and frequency (percentage) for categorical variables. Between-group comparisons were performed using the independent two sample t-test for continuous variables and the chi‐squared or Fisher’s exact test for categorical variables as appropriate. The mean imputation for missing value of laboratory findings was performed to minimize the sample size loss. The cumulative incidence rate of clinical endpoints was estimated using the Kaplan–Meier method and compared between groups using the log-rank test. Cox proportional hazard regression analyses were performed to calculate hazard ratio (HR) with 95% confidence interval (CI) for each clinical endpoint associated with IVUS- or angiography-guided PCI. Landmark analysis was conducted at 30 days after PCI. Inverse probability of treatment weighting (IPTW) was used to adjust for confounding factors. The propensity score (PS) was estimated using multiple logistic regression analysis with all covariates. The standardized mean difference (SMD) was used to assess the balance of covariate distribution between the groups. Covariates with an SMD < 0.1 were considered balanced. The cumulative incidence rate of clinical endpoints was calculated using the IPTW-adjusted Kaplan–Meier estimators and compared between groups using IPTW-adjusted log-rank test. We also conducted sensitivity analyses using PS matching to enhance the validity of our results. The IVUS and angiography groups were matched in a 1:2 ratio without replacement using the nearest-neighbor method based on a PS with a 0.1-caliper width. Additionally, we performed univariable and multivariable logistic regression analyses to identify the determinants for IVUS use. Significant variables (i.e., P < 0.10) in the univariable analysis were included in the multivariable analysis. All statistical analyses were conducted using SAS version 9.3 (SAS Institute, Cary, NC, USA) and R software (version 4.1.1; R Foundation for Statistical Computing, Vienna, Austria). P < 0.05 was considered significant.

## Results

### Baseline characteristics

The baseline characteristics are summarized in Tables 1 and 2. Overall, 1418 patients were evaluated: 294 (20.7%) underwent IVUS-guided PCI, and 1124 (79.3%) underwent angiography-guided PCI with second-generation DES implantation. The mean age was 66.6 ± 12.3 years, and 75.1% of the patients were men. In total, 1138 patients (80.3%) presented with ST-segment elevation myocardial infarction (STEMI). The IVUS-guided PCI group was younger and had a higher frequency of prior MI history. The left ventricular ejection fraction was similar between the groups. Although the proportion of multivessel disease was higher in the IVUS-guided PCI group, the proportion of patients treated with culprit-only PCI strategy among patients with multivessel disease was not significantly different. Regarding the lesion profiles, the proportion of culprit vessels located in the left main artery and prevalence of type B2/C lesion were higher in the IVUS-guided PCI group. During the procedure, 18.2% of patients required hemodynamic support devices. The proportion of transradial approach and use of glycoprotein IIb/IIIa inhibitors were higher in the IVUS-guided PCI group. The IVUS-guided PCI group showed a higher number of implanted stents (1.33 ± 0.55 vs. 1.16 ± 0.40; p < 0.01), larger implanted stent diameter (3.27 ± 0.51 mm vs. 3.16 ± 0.43 mm; p < 0.01), and longer implanted stent (34.9 ± 18.7 mm vs. 30.3 ± 14.2 mm; p < 0.01). After PS matching and IPTW adjustment, the standardized differences between the groups were < 10.0% for all variables, indicating appropriate matching (Supplementary Tables S1 and S2).

**Table 1:** Baseline characteristics.

Characteristics	Crude population				IPTW population		
	IVUS-guided (n = 294)	Angiography-guided (n = 1124)	P value	SMD	IVUS-guided (n = 294)	Angiography-guided (n = 1124)	SMD
Demographics, n (%)							
Age, y, mean (SD)	64.7 ± 12.4	67.1 ± 12.2	< 0.01	0.19	66.7 ± 12.2	66.7 ± 12.3	0.01
Male gender	227 (77.2)	838 (74.6)	0.36	0.06	213 (72.4)	839 (74.7)	0.05
BMI, median (IQR)	24.1 ± 3.6	23.7 ± 3.2	0.08		23.7 ± 3.3	23.7 ± 3.0	0.02
Clinical presentation			0.06	0.13			0.03
STEMI	224 (76.2)	914 (81.3)			232 (79.0)	903 (80.4)	
NSTEMI	70 (23.8)	210 (18.7)			62 (21.0)	221 (19.6)	
Cardiovascular risk factors, n (%)							
Hypertension	162 (55.1)	601 (53.5)	0.65	0.03	159 (54.1)	606 (53.9)	< 0.01
Diabetes mellitus	85 (28.9)	376 (33.5)	0.14	0.10	89 (30.1)	365 (32.5)	0.05
Dyslipidemia	35 (11.9)	129 (11.5)	0.84	0.01	34 (11.5)	131 (11.6)	< 0.01
Current smoker	122 (41.5)	413 (36.7)	0.13	0.10	115 (39.0)	421 (37.5)	0.03
Prior MI	29 (9.9)	63 (5.6)	0.01	0.16	19 (6.6)	74 (6.6)	< 0.01
Prior revascularization	8 (2.7)	48 (4.3)	0.31	0.08	9 (3.2)	44 (3.9)	0.04
Prior CVA	15 (5.1)	96 (8.5)	0.051	0.14	24 (8.1)	89 (7.9)	0.01
LVEF, median (IQR)	49.0 ± 12.00	48.2 ± 11.7	0.28	0.07	48.8 ± 11.6	48.4 ± 11.7	0.04
Laboratory findings							
eGFR	63.0 ± 27.3	64.1 ± 41.4	0.68	0.03	62.6 ± 26.4	63.8 ± 38.9	0.04
Peak CK-MB, μg/L	177.5 ± 190.9	176.7 ± 213.0	0.95	< 0.01	179.1 ± 189.1	177.5 ± 211.3	0.01
LDL-cholesterol, mg/dL	101.6 ± 37.9	100.0 ± 40.2	0.54	0.04	102.4 ± 39.0	100.4 ± 40.5	0.05
CRP, mg/L	2.3 ± 3.6	2.8 ± 4.9	0.053	0.12	2.5 ± 3.7	2.7 ± 4.7	0.05
Discharge medication							
DAPT	272 (92.5)	972 (86.5)	0.01	0.20	262 (89.0)	985 (87.7)	0.04
Aspirin	273 (92.9)	982 (87.4)	< 0.01	0.18	263 (89.3)	994 (88.4)	0.03
P2Y_12_ inhibitor			0.08	0.26			0.05
Clopidogrel	144 (49.0)	585 (52.0)			150 (51.2)	575 (51.2)	
Ticagrelor	111 (42.2)	325 (33.0)			94 (32.1)	346 (30.8)	
Prasugrel	19 (6.5)	70 (6.2)			18 (6.2)	70 (6.2)	
ACEi or ARB	164 (55.8)	662 (58.9)	0.34		168 (57.3)	655 (58.3)	0.02
Beta-blocker	198 (67.3)	680 (60.5)	0.03		179 (60.9)	693 (61.6)	0.01
Statin	250 (85.0)	855 (76.1)	< 0.01		227 (77.1)	874 (77.4)	0.01

Data are presented as mean (SD), median (interquartile range), or n (%).

**Table 2:** Lesion and procedural characteristics.

Characteristics	Crude population				IPTW population		
	IVUS-guided (n = 294)	Angiography-guided (n = 1124)	P value	SMD	IVUS-guided (n = 294)	Angiography-guided (n = 1124)	SMD
Lesion characteristics, n (%)							
Multivessel disease	194 (66.0)	651 (57.9)	0.01	0.17	175 (59.5)	668 (69.5)	< 0.01
Revascularization in index PCI			0.45	0.12			0.01
Culprit only	120 (61.9)	430 (66.1)			132 (67.9)	426 (65.5)	
Multivessel PCI	74 (38.1)	219 (33.6)			62 (32.1)	223 (34.3)	
Culprit vessel			0.12	0.16			0.08
Left main artery	24 (8.2)	59 (5.2)			17 (5.8)	66 (5.9)	
LAD	105 (35.7)	362 (32.2)			104 (35.4)	369 (32.8)	
LCX	28 (9.5)	130 (11.6)			32 (12.7)	127 (11.3)	
RCA	137 (46.6)	573 (51.0)			136 (46.1)	562 (50.0)	
ACC/AHA B2/C lesion	267 (90.8)	953 (84.8)	< 0.01	0.19	256 (87.1)	967 (86.0)	0.03
Procedural characteristics, n (%)							
Trans-radial approach	119 (41.9)	329 (30.2)	< 0.01	0.24	91 (30.8)	353 (31.4)	0.01
Glycoprotein IIb/IIIa inhibitor	60 (20.4)	147 (13.1)	< 0.01	0.20	45 (15.2)	163 (14.5)	0.02
Thrombus aspiration	52 (18.5)	234 (21.5)	0.25	0.08	63 (21.4)	227 (20.2)	0.03
Stent type			0.77	0.10			0.05
Zotarolimus	62 (21.1)	232 (20.6)			60 (20.2)	233 (20.8)	
Everolimus	157 (53.4)	582 (51.8)			149 (50.8)	585 (52.0)	
Sirolimus	41 (13.9)	146 (13.0)			38 (13.1)	148 (13.2)	
Biolimus	25 (8.5)	115 (10.2)			34 (11.5)	112 (10.0)	
Novolimus	9 (3.1)	49 (4.4)			13 (4.4)	46 (4.1)	
Successful PCI	287 (97.6)	1,105 (98.3)	0.46	0.05	287 (97.7)	1104 (98.3)	0.04
Multiple stent implantation	84 (28.6)	161 (14.3)	< 0.01	0.35	51 (17.4)	191 (17.0)	0.01
Stent length ≥ 60 mm	30 (10.2)	65 (5.8)	0.01	0.27	19 (6.6)	76 (6.7)	< 0.01
Mechanical support	51 (17.3)	207 (18.4)	0.73	0.03	59 (20.0)	201 (17.9)	0.05
IABP	33 (11.2)	147 (13.1)	0.43	0.06	40 (13.7)	143 (12.8)	0.03
ECMO	19 (6.5)	91 (8.1)	0.39	0.06	17 (5.7)	86 (7.7)	0.08
In-hospital course							
In-hospital CPR	90 (30.6)	351 (31.2)	0.89	0.01	87 (29.8)	348 (31.0)	0.03
Length of hospital stays	9.1 ± 16.6	8.8 ± 21.4	0.77	0.02	8.4 ± 12.5	8.8 ± 21.5	0.03

Data are presented as the mean (SD), or n (%).

### One-year clinical outcomes

**Figure 2:**
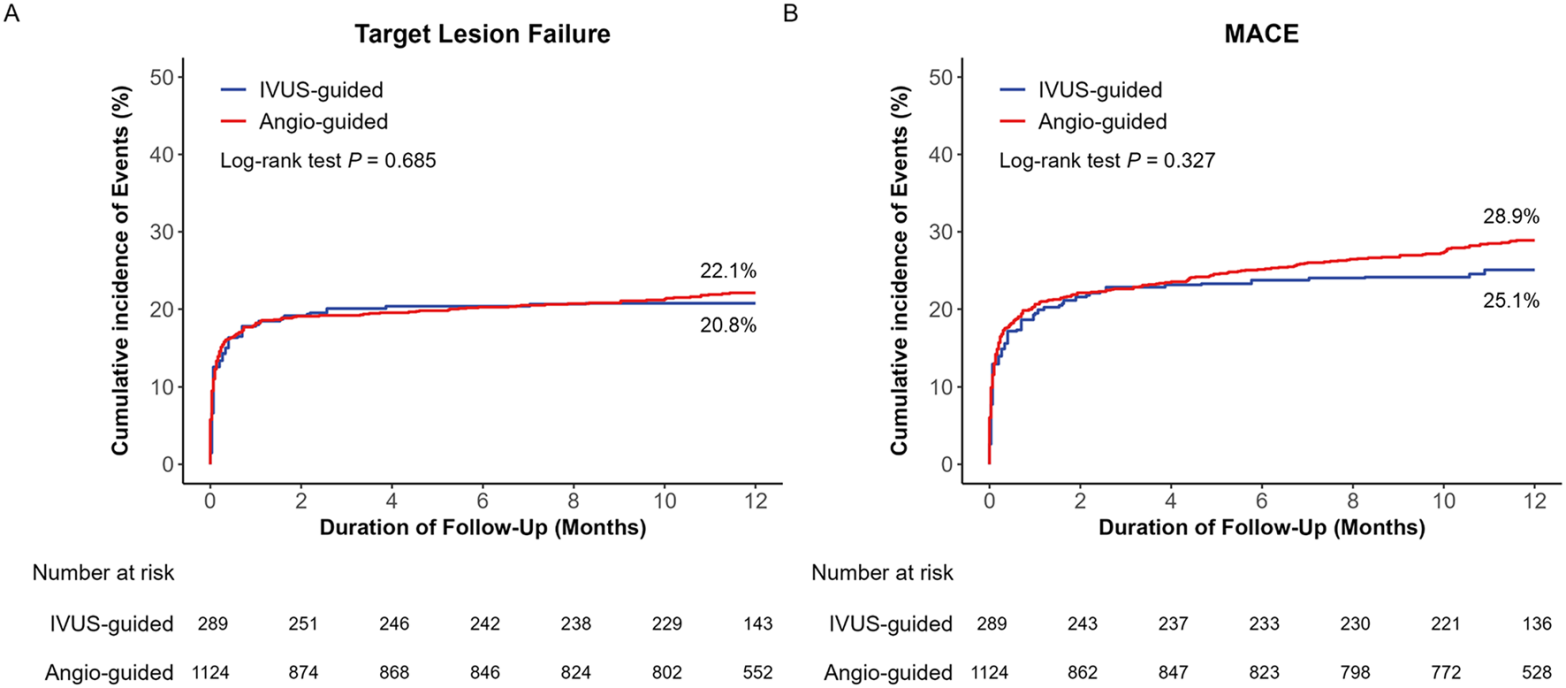
Kaplan–Meier of the rate of 1-year target lesion failure (TLF) and major adverse cardiac events (MACE) in IVUS-guided and angiography-guided PCI. TLF (**A**) and MACE (**B**). IPTW-adjusted log-rank p values are presented inside each panel.

Fig. 2, Table 3, and Supplementary Fig. S3 present the comparison of 1-year clinical outcomes between the IVUS- and angiography-guided PCI groups. The median follow-up duration was 360 days (interquartile range, 284–382 days). Overall, 311 TLFs (21.9%; 286 cardiac deaths, 7 TV-MI, and 27 ID-TLRs) occurred during the 1-year follow-up. The risk of TLF at 1 year was significantly lower in the IVUS-guided PCI group, but there was no difference after multiple sensitivity analyses (multivariable adjusted HR: 0.74; 95% CI 0.54–1.05; p = 0.10, PS-matched HR: 0.86; 95% CI 0.57–1.29; p = 0.47, and IPTW-adjusted HR: 0.93; 95% CI 0.65–1.34; p = 0.70). Regarding the secondary outcomes, risk of MACE, all-cause death, and cardiac death did not significantly differ between the two groups after adjustment, although the unadjusted analyses showed significantly lower rates in the IVUS‐guided PCI group. There was no significant between-group difference in the risk of any MI, TV-MI, any revascularization, ID-TLR, and definite/probable stent thrombosis. The risk of TLF and MACE were comparable between the groups in both patients with STEMI and with NSTEMI (Supplementary Tables S3 and S4). No significant interaction was observed in the subgroup analyses (Supplementary Fig. S4).

**Table 3:** One-year outcome.

	IVUS-guided (n = 294)	Angio-guided (n = 1124)	Unadjusted		Multivariable-adjusted^a^		PS-matched		IPTW-adjusted	
			Hazard ratio (95% CI)	*p* value	Hazard ratio (95% CI)	*p* value	Hazard ratio (95% CI)	*p* value	Hazard ratio (95% CI)	*p* value
Primary outcome										
Target lesion failure	48 (16.3)	263 (23.4)	0.66 (0.48–0.89)	< 0.01	0.74 (0.54–1.05)	0.10	0.86 (0.57–1.29)	0.47	0.93 (0.65–1.34)	0.70
Secondary outcome										
MACE	64 (21.8)	349 (31.0)	0.66 (0.51–0.87)	< 0.01	0.76 (0.57–1.01)	0.06	0.83 (0.59–1.18)	0.31	0.86 (0.63–1.18)	0.36
All-cause death	49 (16.7)	286 (25.4)	0.61 (0.45–0.83)	< 0.01	0.71 (0.51–0.98)	0.04	0.85 (0.58–1.26)	0.43	0.89 (0.62–1.27)	0.51
Cardiac death	43 (14.6)	243 (21.6)	0.64 (0.46–0.88)	< 0.01	0.76 (0.53–1.09)	0.13	0.93 (0.61–1.41)	0.74	0.98 (0.68–1.43)	0.92
Any MI	10 (3.4)	20 (1.8)	1.86 (0.87–3.99)	0.11	1.32 (0.53–3.30)	0.55	2.00 (0.58–6.91)	0.27	1.44 (0.63–3.29)	0.39
TV-MI	3 (1.0)	4 (0.4)	2.60 (0.58–11.63)	0.21	NA	NA	1.00 (0.09–11.03)	1.00	1.16 (0.24–5.59)	0.85
Any revascularization	15 (4.8)	64 (5.5)	0.81 (0.45–1.45)	0.48	0.70 (0.37–1.31)	0.26	0.68 (0.30–1.55)	0.36	0.70 (0.37–1.33)	0.28
ID-TLR	6 (1.7)	21 (1.7)	0.92 (0.34–2.47)	0.87	0.58 (0.19–1.77)	0.34	0.50 (0.11–2.35)	0.38	0.51 (0.18–1.43)	0.20
Definite/probable ST	5 (1.7)	13 (1.2)	1.43 (0.51–4.02)	0.50	1.43 (0.51–4.04)	0.50	1.50 (0.34–6.70)	0.60	1.27 (0.43–3.75)	0.67

Data are presented as the mean (SD), or n (%).

^a^The confounding factors considered in the adjusted hazard ratio are age, sex, BMI, clinical presentation, hypertension, diabetes mellitus, dyslipidemia, current smoker, prior MI, prior revascularization, prior CVA, LVEF, eGFR, CK-MB, LDL-choleterol, CRP, DAPT, aspirin, P2Y12 inhibitor, ACEi or ARB, beta-blocker, statin, multi-vessel disease, culprit vessel, B2/C lesion, trans-radial approach, glycoprotein IIb/IIIa inhibitor, thrombus aspiration, thrombus type, successful PCI, multiple stent implatnation, stent length ≥ 60 mm, IABP, ECMO, in-hospital CPR and length of hospital stays.

### Thirty-day outcome and landmark analysis

**Figure 3:**
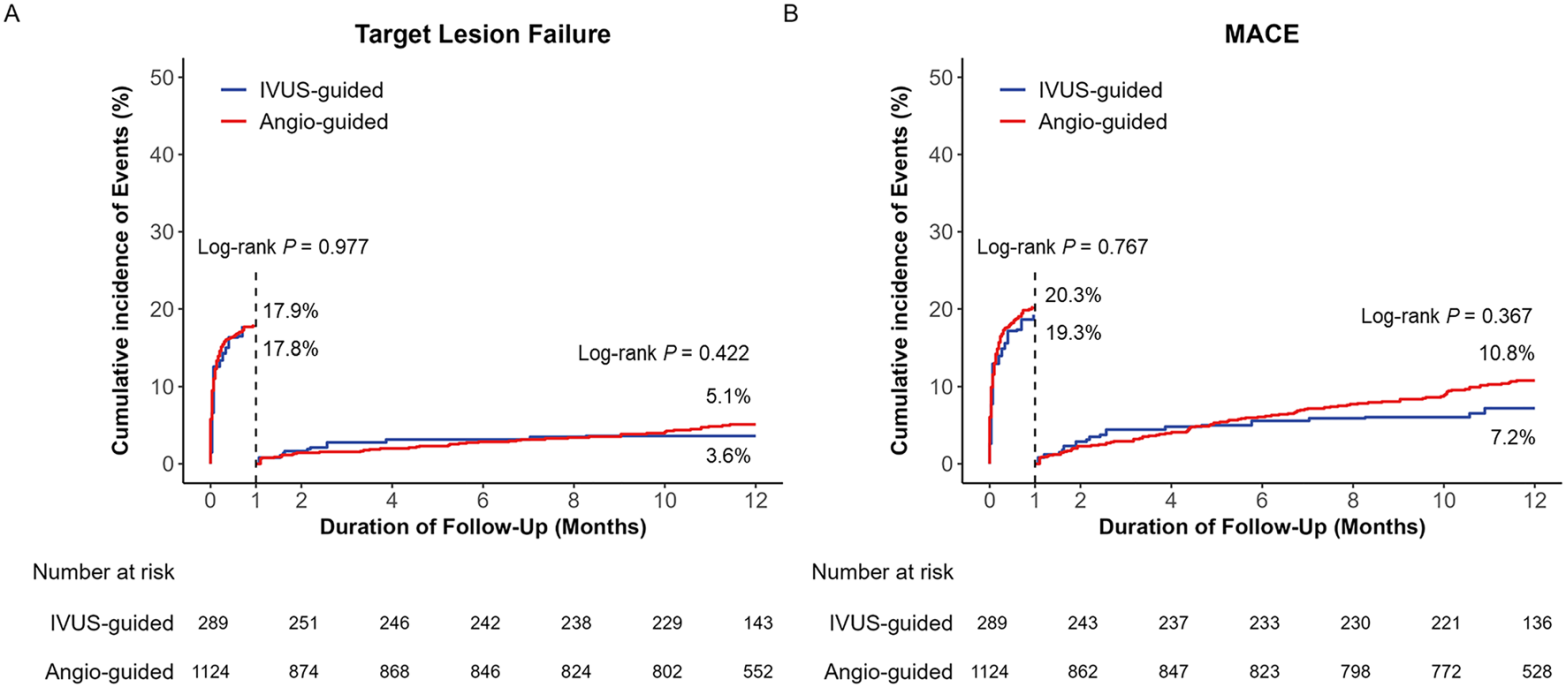
Landmark analysis for target lesion failure (TLF) and major adverse cardiac events (MACE) before and after 30-days of follow-up. TLF (**A**) and MACE (**B**). IPTW-adjusted log-rank p values are presented inside each panel.

Supplementary Tables S5 and S6, and Fig. 3 present the clinical outcomes at 30 days and between 30 days and 1 year of follow-up in the IVUS- and angiography-guided PCI groups. Regarding short-term clinical outcomes, although the unadjusted rates for 30-day TLF and MACE were significantly lower in the IVUS‐guided PCI group, multivariable, PS matching, and IPTW adjustment revealed that the risks of 30-day TLF and MACE, all-cause death, and cardiac death did not differ significantly between the two groups. TLF and MACE also did not differ before and after multiple sensitivity analyses.

### Major factors influencing IVUS usage

**Figure 4:**
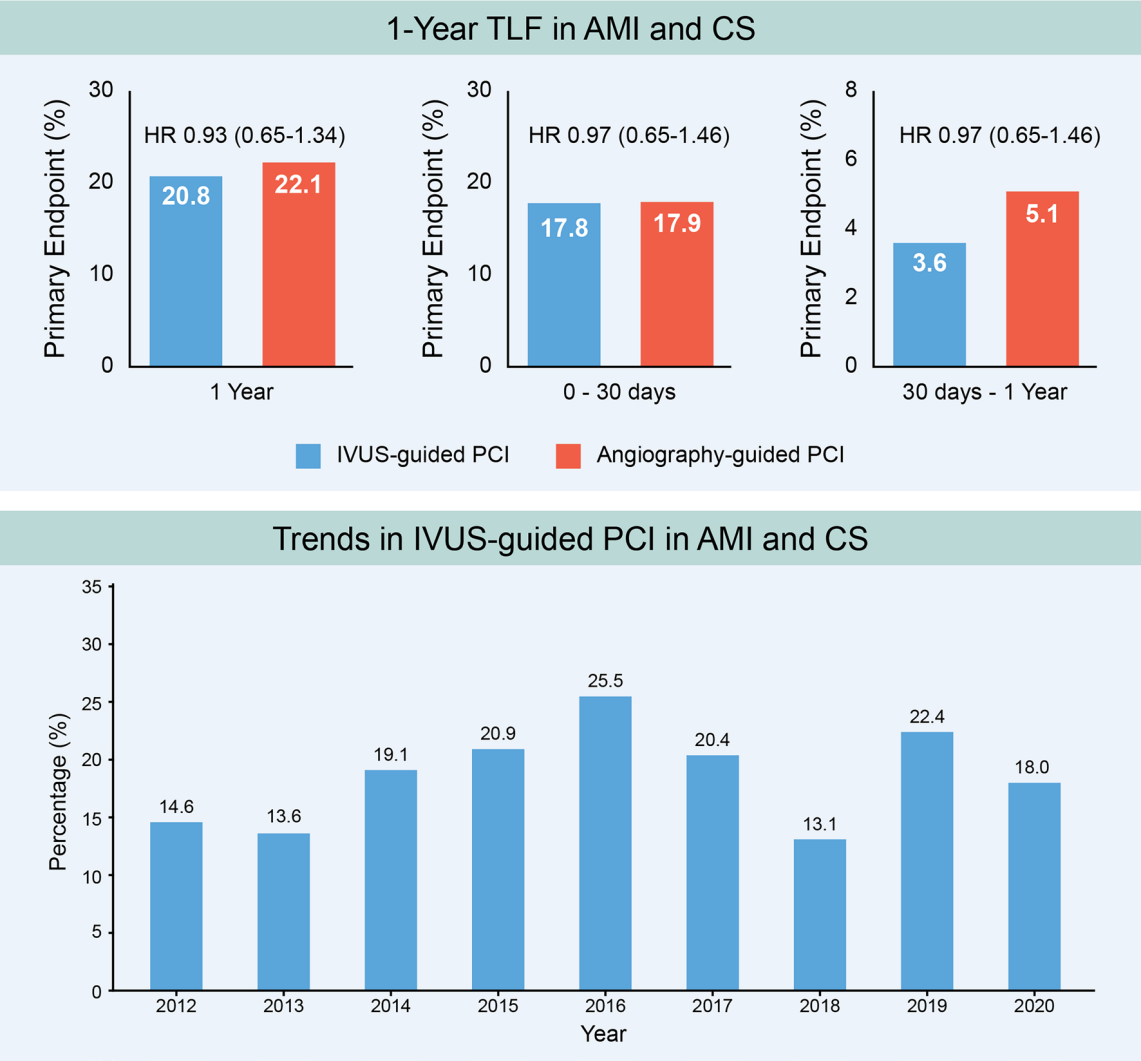
Intravascular ultrasound versus angiography-guided percutaneous coronary intervention in patients with acute myocardial infarction and cardiogenic shock.

In total, 7.2% (2095/28,949) patients were treated for CS complicating AMI between 2011 and 2020. Among them, 18.6% (354/1907) of patients underwent PCI with IVUS guidance, and the remaining 81.4% (1553) underwent PCI with angiography guidance. Among patients with AMI accompanied by CS, the rate of IVUS utilization has consistently remained over 10% (Fig. 4) in patients who underwent PCI. The primary factors for IVUS usage were younger patients (aged < 65 years), prior MI, left main disease, and multiple stent implantation (Table 4). Although not significant, there was a trend towards higher IVUS utilization in patients with NSTEMI than in patients with STEMI.

**Table 4:** Univariable and multivariable analyses on the determinants for IVUS usage.

Variables	Univariable analysis		Multivariable analysis	
	Odds ratio (95% CI)	P value	Odds ratio (95% CI)	P value
Clinical variables				
Age < 65 years	1.38 (1.07–1.79)	0.01	1.52 (1.16–1.98)	< 0.01
Male gender	1.16 (0.86–1.58)	0.35		
NSTEMI	1.36 (1.00–1.84)	0.05	1.34 (0.97–1.83)	0.07
Diabetes mellitus	0.81 (0.61–1.07)	0.14		
CKD (eGFR < 60)	0.81 (0.62–1.05)	0.11		
Prior MI	1.84 (1.15–2.89)	< 0.01	1.98 (1.22–3.17)	< 0.01
Prior revascularization	0.63 (0.27–1.27)	0.23		
LVEF < 50%	0.81 (0.63–1.05)	0.11		
Angiographic variables				
Multi-vessel CAD	1.41 (1.08–1.85)	0.01	1.21 (0.91–1.60)	0.2
LM disease	2.03 (1.31–3.10)	< 0.01	1.98 (1.25–3.09)	< 0.01
Procedural variables				
IABP	0.84 (0.55–1.24)	0.40		
ECMO	0.78 (0.46–1.28)	0.35		
Multiple stent implantation	2.39 (1.76–3.23)	< 0.01	2.69 (1.85–3.90)	< 0.01
Stent diameter ≥ 3 mm	1.21 (0.89–1.67)	0.23		
Stent length ≥ 60 mm	1.85 (1.16–2.89)	< 0.01	0.81 (0.46–1.40)	0.45

## Discussion

The main findings of the present study were as follows (Central Illustration). First, there was no difference in 1-year TLF risk between the IVUS- and angiography-guided PCI groups after adjustment for confounding factors. Second, the adjusted landmark analysis for TLF and MACE showed no significant differences between the two groups at within 30 days and between 30 days and 1 year. Third, the major factors for IVUS usage were younger patients, prior MI, left main disease, and multiple stent implantation.

IVUS-guided PCI is associated with better clinical outcomes than angiography-guided PCI in the second-generation DES era^15,16^. However, previous studies excluded patients with CS. Two AMI registries demonstrated that IVUS-guided PCI improved long-term clinical outcomes compared with angiography-guided PCI in real-world practice. However, one study using the KAMIR-NIH registry excluded patients with CS, whereas in another AMI registry, only approximately 8.7% (855/9846) of the patients had CS. Furthermore, no further subgroup analysis was conducted for patients with CS^8,9^. The role of intravascular imaging during revascularization is also not mentioned in the current guideline for the management of CS complicating MI^17^. Therefore, the importance of this study lies in its primary focus on comparing clinical outcomes between IVUS and angiography guidance in patients with CS derived from a dedicated AMI registry.

In this study, IVUS was consistently utilized in PCI procedures for 13–25% of all patients diagnosed with AMI and CS. The unusually high rate of IVUS penetration in the clinical context of AMI and CS might be attributed to several factors. First, the positive outcomes observed in randomized trials and dedicated AMI registries with IVUS guidance may have influenced the operators to choose PCI optimization even in patients with AMI and CS. Furthermore, the presence of left main disease was associated with IVUS utilization in the current study. Because current guidelines recommend IVUS guidance for left main-PCI, the operators may have faithfully adhered to this guideline in cases of CS complicating AMI^18^. Second, IVUS guidance was selected for patients with a history of MI and those undergoing multiple stent implantation, as they are considered as having high risk for subsequent ischemic events. Therefore, IVUS for these patients is aimed to minimize the risk of future ischemic events. Third, physicians seemed to favor IVUS-guided PCI among relatively young patients (age < 65 years), anticipating its long-term benefits in reducing the risk of ischemic event. Indeed, IVUS-guided PCI consistently reduced ischemic risks even beyond the 1-year follow up^19^. However, IVUS-guided PCI in patients with AMI and CS in the present study did not improve TLF reflecting ischemic events compared with angiography-guided PCI in the same population. In the landmark analysis, IVUS-guided PCI also did not reduce ischemic risk between 30 days and 1 year after the index procedure compared with angiography-guided PCI. Notably, the IVUS group showed an equal TLR rate to that of angiography alone despite the greater complexity of the PCI procedure. These results may offer valuable insights for physicians in prioritizing treatment strategies in the challenging scenario of CS complicating AMI. Nevertheless, the benefit of IVUS use in particular patients, such as those with distal lesions of the left main artery of with confusing angiographic findings, cannot be excluded.

Given the substantial predictive value of CS in relation to stent thrombosis^20^, physicians have made efforts to optimize stent deployment under IVUS guidance, aiming to minimize acute or subacute ischemic events. However, adjusted landmark analysis from the present study showed no difference in 30-day clinical outcomes between the IVUS- and angiography-guided PCI groups. In the future, a well-designed, large scale randomized trial is needed to identify a specific patient subgroup in whom IVUS-guided PCI could enhance the clinical outcomes for individuals with AMI and CS.

## Limitations

First, the non-randomized observational design introduced inherent selection and information biases. Using physician discretion to determine treatment strategy inevitably introduced the possibility of selection bias. Furthermore, the number of patients was largely different between the groups. Although, extensive sensitivity analyses were conducted to adjust for the measured or unmeasured confounding factors to minimize the bias from different baseline characteristics, the possibility of unmeasured confounders influencing the findings cannot be excluded. Second, despite the pooled analyses, differences between centers and operator’s experiences on IVUS may affect the findings. Third, the decision to use IVUS was made at the operator’s discretion. Fourth, detailed procedural data were missing. Additionally, we did not have the timing of intravascular imaging relative to the PCI procedure. Therefore, the use of IVUS-guided PCI did not guarantee the optimization of PCI, and the findings should be interpreted cautiously. Fifth, detailed procedural data procedure time, and total amount of contrast media were unavailable. Finally, procedure-related risks were not evaluated.

In conclusion, IVUS-guidance did not improve the 1-year TLF compared with angiography-guidance in patients with AMI and CS who underwent PCI with second-generation DES. Further, risk of TLF at 30 days and between 30 days and 1 year after PCI were also comparable between the two groups. The factors for IVUS usage were younger age, prior MI, LM coronary artery disease, and multiple stent implantation.

### Supplementary Information

Supplementary Information.

## Data Availability

The datasets used and/or analysed during the current study available from the corresponding author on reasonable request.
