# Supplementary material for: Intravascular ultrasound-guided versus angiography-guided percutaneous coronary intervention for acute myocardial infarction with cardiogenic shock
Source: Sci Rep. 2024 May 1;14:10028. doi: 10.1038/s41598-024-59723-y (PMC11063208; doi:10.1038/s41598-024-59723-y)
Supplement: Supplementary file 1 — Supplementary Information. [file 41598_2024_59723_MOESM1_ESM.docx]

**Supplementary Information**

**
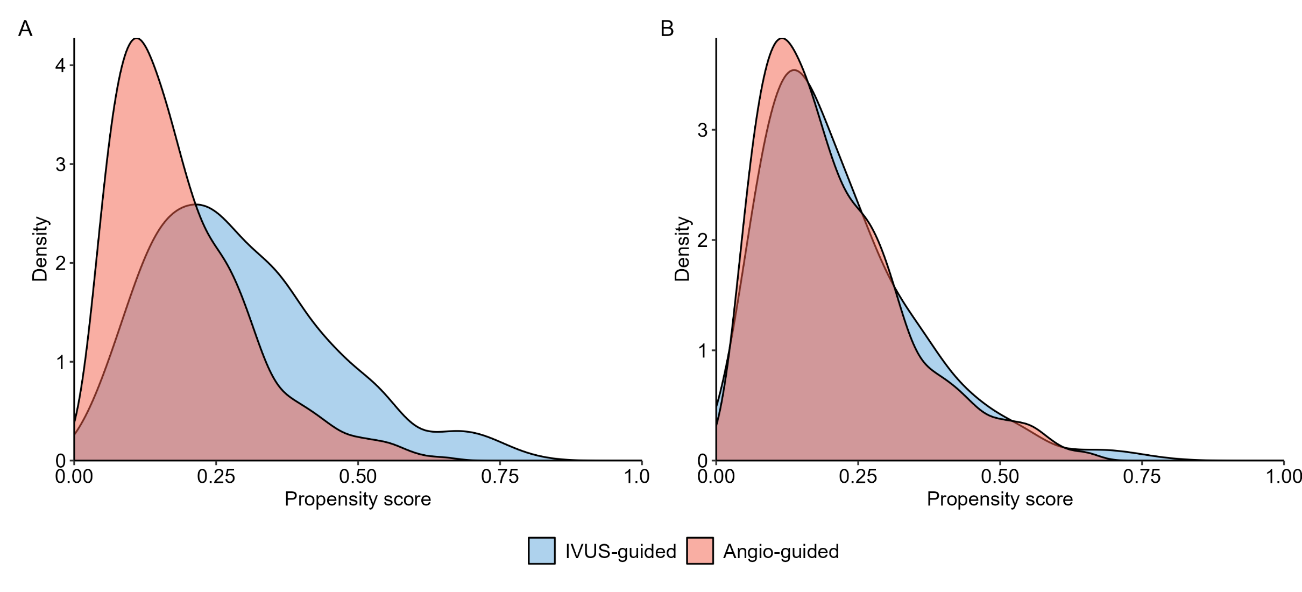
**

**Supplementary Fig. S1.** Propensity score distribution before and after IPTW adjustment. IVUS, intravascular ultrasound.

**
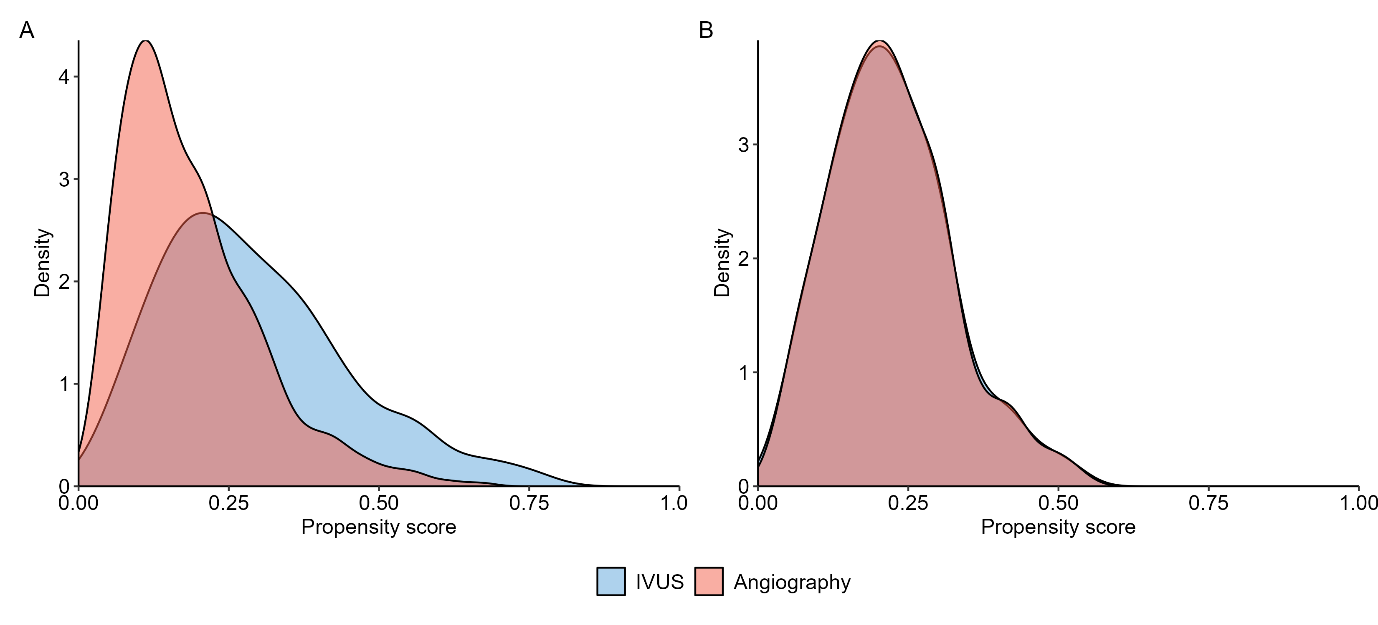
**

**Supplementary Fig. S2.** Propensity score distribution before and after PS matching.
IVUS, intravascular ultrasound.


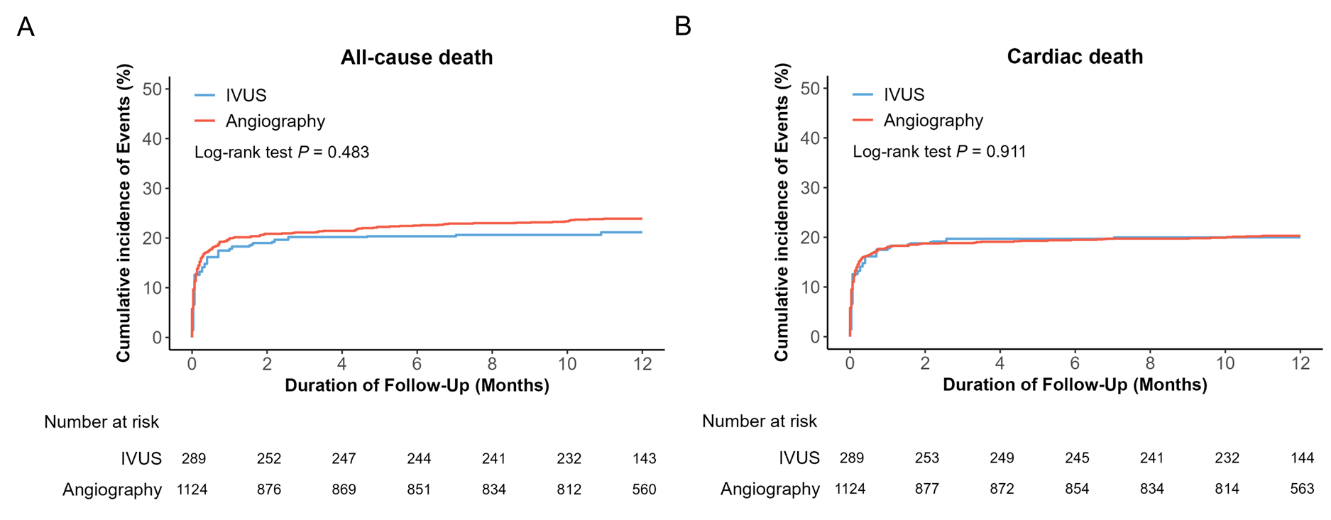

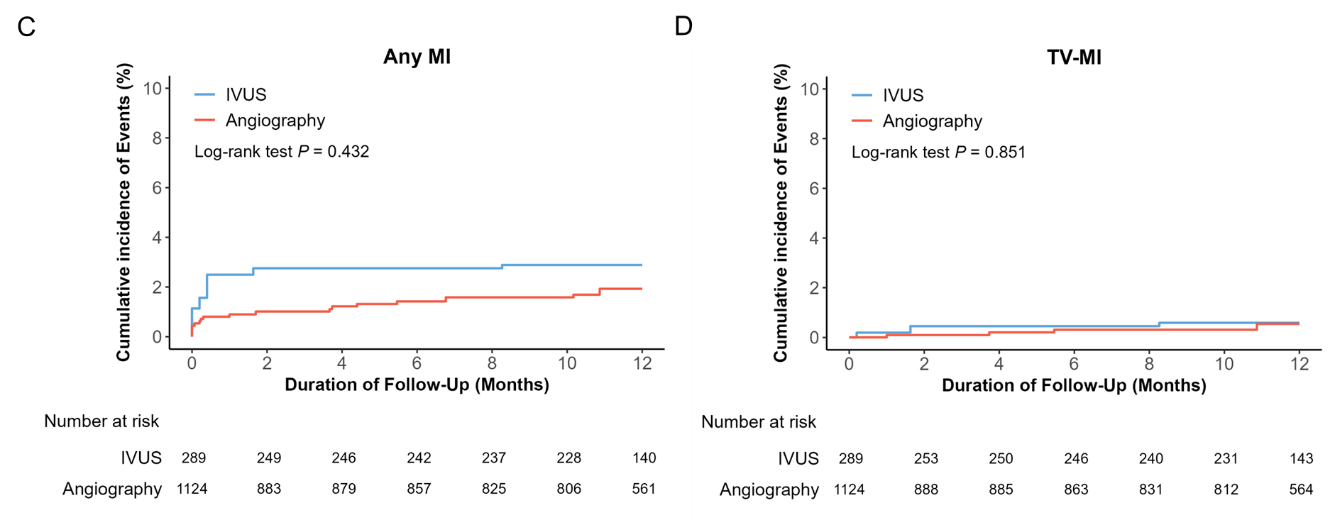


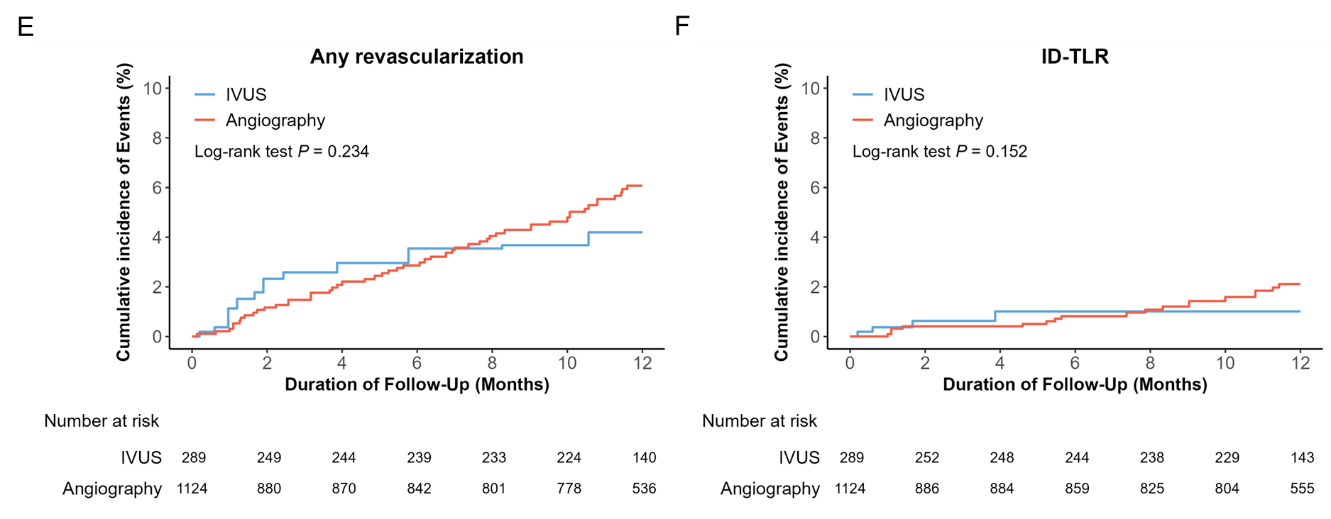


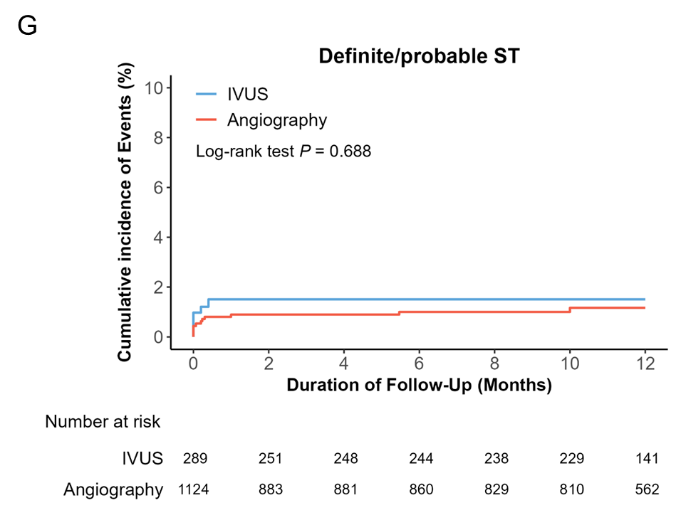


**Supplementary Fig. S3.** Kaplan-Meier curves for 1-year all-cause death, cardiac death, all MI, TV-MI, any revascularization, ID-TLR and stent thrombosis. All-cause death (**A**), cardiac death (**B**), any MI (**C**), TV-MI (**D**), any revascularization (**E**), ID-TLR (**F**), and definite/probable ST (**G**). IPTW-adjusted log-rank p values are presented inside each panel. IPTW, inverse probability of treatment weighting; ID-TLR, ischemia driven-target lesion revascularization; IVUS, intravascular ultrasound; MI, myocardial infarction; ST, stent thrombosis; TV-MI, target vessel myocardial infarction.


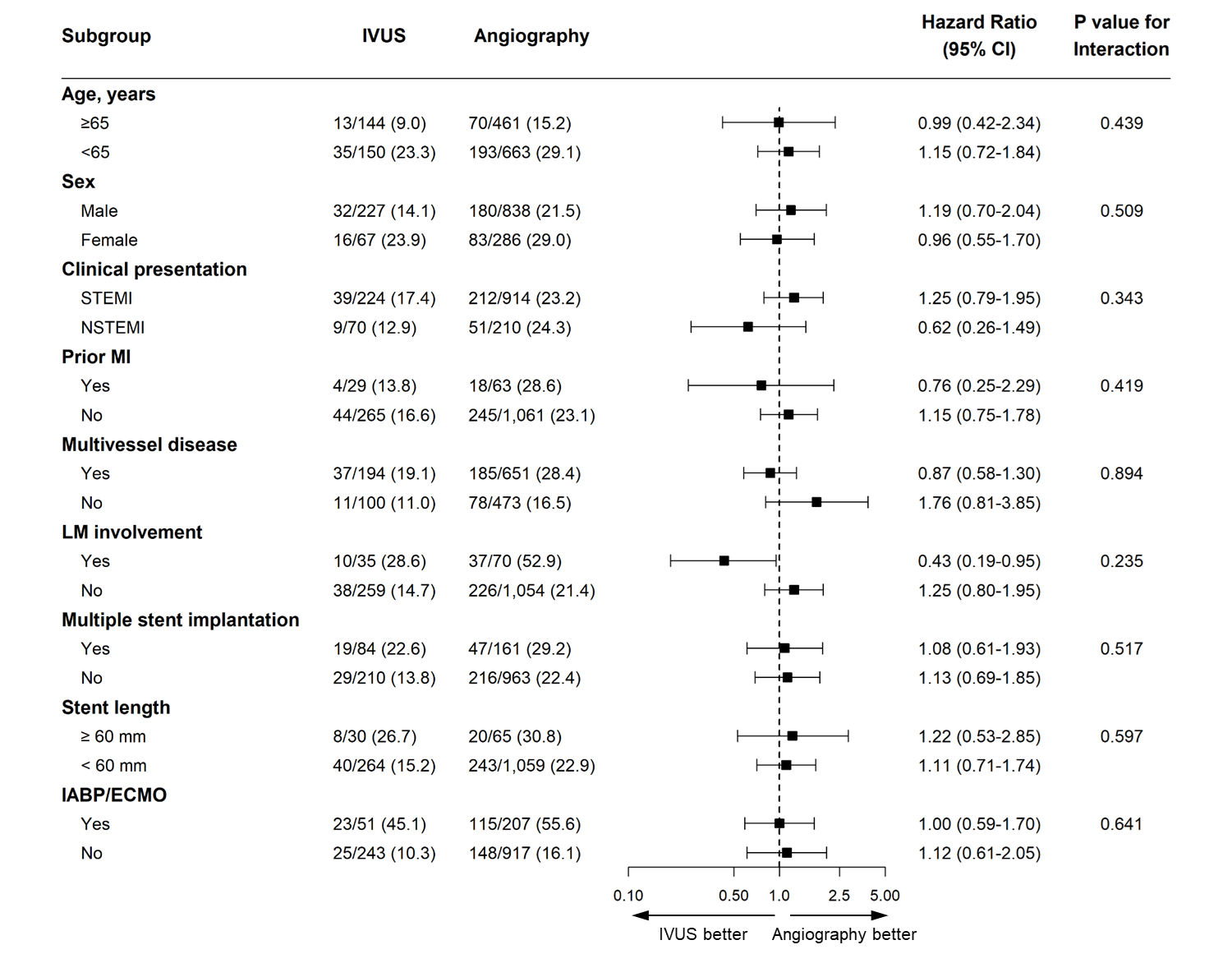


**Supplementary Fig. S4.** Subgroup analysis for 1-year target lesion failure (TLF). ECMO, extracorporeal membrane oxygenation; IABP, intra-aortic balloon pump; IVUS, intravascular ultrasound; LM, left main artery; MI, myocardial infarction; NSTEMI, non-ST-segment elevation myocardial infarction; STEMI, ST-segment elevation myocardial infarction

**Supplementary Tables**

**Supplementary Table S1. baseline characteristics**

| **Characteristics** | **PS-matched population** | | |  |
| --- | --- | --- | --- | --- |
|  | **IVUS-guided (n=212)** | **Angiography-guided (n=424)** | **SMD** |  |
| **Demographics, n (%)** |  |  |  |  |
| Age, y, mean (SD) | 66.2±12.3 | 65.4±12.8 | 0.06 |  |
| Male gender | 154 (72.6) | 309 (72.9) | 0.01 |  |
| BMI, median (IQR) | 24.0±3.6 | 23.9±3.2 | 0.03 |  |
| **Clinical presentation** |  |  | 0.01 |  |
| STEMI | 167 (78.8) | 333 (78.5) |  |  |
| NSTEMI | 45 (21.2) | 91 (21.5) |  |  |
| **Cardiovascular risk factors, n (%)** | | |  | |
| Hypertension | 118 (55.7) | 229 (54.0) | 0.03 |  |
| Diabetes mellitus | 65 (30.7) | 124 (29.2) | 0.03 |  |
| Dyslipidemia | 22 (10.4) | 43 (10.1) | 0.01 |  |
| Current smoker | 83 (39.2) | 176 (41.5) | 0.05 |  |
| Prior MI | 16 (7.5) | 28 (6.6) | 0.04 |  |
| Prior revascularization | 7 (3.3) | 15 (3.5) | 0.01 |  |
| Prior CVA | 14 (6.6) | 21 (5.0) | 0.07 |  |
| LVEF,   median (IQR) | 49.2±11.6 | 49.0±11.4 | 0.02 |  |
| **Laboratory findings** |  |  |  |  |
| eGFR | 63.3±27.0 | 64.8±30.1 | 0.05 |  |
| Peak CK-MB, μg/L | 181.1±208.1 | 178.1±226.2 | 0.01 |  |
| LDL-cholesterol   ,mg/dL | 100.4±37.0 | 103.0±49.6 | 0.06 |  |
| CRP, mg/L | 2.4±3.5 | 2.4±3.9 | 0.01 |  |
| **Discharge medication** | |  |  |  |
| DAPT | 193 (91.0) | 389 (91.7) | 0.03 |  |
| Aspirin | 194 (91.5) | 390 (92.0) | 0.02 |  |
| P2Y_12_ inhibitor |  |  | 0.04 |  |
| Clopidogrel | 109 (51.4) | 217 (51.2) |  |  |
| Ticagrelor | 72 (34.0) | 148 (34.9) |  |  |
| Prasugrel | 13 (6.1) | 27 (6.4) |  |  |
| ACEi or ARB | 127 (59.9) | 263 (62.0) | 0.04 |  |
| Beta-blocker | 134 (63.2) | 286 (67.5) | 0.09 |  |
| Statin | 174 (82.1) | 351 (82.8) | 0.02 |  |

Data are presented as mean (SD), median (interquartile range), or n (%).

**Supplementary Table S2. Lesion and procedural characteristics**

| **Characteristics** | **PS-matched population** | | | |
| --- | --- | --- | --- | --- |
|  | **IVUS-guided (n=212)** | **Angiography-guided (n=424)** | **SMD** | |
| **Lesion characteristics, n (%)** | | |  |  |
| Multivessel disease | 131 (60.1) | 279 (64.0) | 0.08 | |
| Revascularization in   index PCI |  |  | 0.05 | |
| Culprit only | 84 (66.1) | 177 (68.6) |  | |
| Multivessel PCI | 43 (33.9) | 81 (31.4) |  | |
| Culprit vessel |  |  | 0.09 | |
| Left main artery | 13 (6.1) | 18 (4.2) |  | |
| LAD | 68 (32.1) | 148 (34.9) |  | |
| LCX | 24 (11.3) | 43 (10.1) |  | |
| RCA | 107 (50.5) | 215 (50.7) |  | |
| ACC/AHA B2/C lesion | 188 (88.7) | 370 (87.3) | 0.04 | |
| **Procedural characteristics, n (%)** | |  |  | |
| Trans-radial approach | 73 (34.4) | 148 (34.9) | 0.01 | |
| Glycoprotein IIb/IIIa   inhibitor | 36 (17.0) | 62 (14.6) | 0.06 | |
| Thrombus aspiration | 39 (18.4) | 83 (19.6) | 0.03 | |
| Stent type |  |  | 0.08 | |
| Zotarolimus | 49 (23.1) | 95 (22.4) |  | |
| Everolimus | 109 (51.4) | 216 (50.9) |  | |
| Sirolimus | 24 (11.3) | 58 (13.7) |  | |
| Biolimus | 22 (10.4) | 38 (9.0) |  | |
| Novolimus | 8 (3.8) | 17 (4.0) |  | |
| Successful PCI | 209 (98.6) | 418 (98.6) | <0.01 | |
| Multiple  stent implantation | 36 (17.0) | 78 (18.4) | 0.04 | |
| Stent length ≥ 60 mm | 16 (7.5) | 30 (7.1) | 0.02 | |
| **Mechanical support** |  |  |  | |
| IABP | 23 (10.8) | 45 (10.6) | 0.01 | |
| ECMO | 12 (5.7) | 24 (5.7) | <0.01 | |
| **In-hospital course** |  |  |  | |
| In-hospital CPR | 58 (27.4) | 133 (31.4) | 0.09 | |
| Length of hospital stays | 9.4±18.8 | 9.6±26.5 | 0.01 | |

Data are presented as the mean (SD), or n (%).

**Supplemental Table S3. One-year clinical outcome in patients with ST-segment elevation myocardial infarction**

|  | IVUS-guided (n=224) | Angiography-guided (n=914) | Unadjusted | | Multivariable-Adjusted^b^ | | PS-Matched | | IPTW-Adjusted | |
| --- | --- | --- | --- | --- | --- | --- | --- | --- | --- | --- |
|  |  |  | **Hazard ratio (95% CI)** | ***p* value** | **Hazard ratio (95% CI)** | ***p* value** | **Hazard ratio (95% CI)** | ***p* value** | **Hazard ratio (95% CI)** | ***p* value** |
| **Primary outcome** |  |  |  |  |  |  |  |  |  |  |
| Target lesion failure^a^ | 39 (17.4) | 212 (23.2) | 0.71 (0.51-1.00) | 0.05 | 0.69 (0.47-1.00) | 0.05 | 0.89 (0.58-1.38) | 0.61 | 0.86 (0.57-1.30) | 0.48 |
| **Secondary outcome** |  |  |  |  |  |  |  |  |  |  |
| MACE† | 49 (21.9) | 275 (30.1) | 0.70 (0.51-0.94) | 0.02 | 0.69 (0.50-0.96) | 0.03 | 0.80 (0.54-1.18) | 0.26 | 0.80 (0.55-1.15) | 0.22 |
| All-cause death | 40 (17.9) | 226 (24.7) | 0.69 (0.49-0.96) | 0.03 | 0.66 (0.45-0.95) | 0.03 | 0.84 (0.55-1.27) | 0.40 | 0.83 (0.56-1.24) | 0.37 |
| Cardiac death | 36 (16.1) | 198 (21.7) | 0.70 (0.49-1.00) | 0.05 | 0.70 (0.47-1.04) | 0.08 | 0.94 (0.60-1.46) | 0.77 | 0.90 (0.59-1.37) | 0.62 |
| Any MI | 6 (2.7) | 12 (1.3) | 2.06 (0.77-5.51) | 0.15 | 2.23 (0.83-6.04) | 0.11 | 2.50 (0.67-9.31) | 0.17 | 1.70 (0.59-4.90) | 0.33 |
| TV-MI | 1 (0.4) | 2 (0.2) | 1.90 (0.17-20.92) | 0.60 | NA | NA | NA | NA | 1.00 (0.09-10.84) | 1.00 |
| Any revascularization | 10 (4.5) | 48 (5.3) | 0.83 (0.42-1.63) | 0.58 | 0.71 (0.34-1.50) | 0.37 | 0.76 (0.28-2.01) | 0.58 | 0.70 (0.33-1.45) | 0.33 |
| ID-TLR | 4 (1.8) | 14 (1.5) | 1.09 (0.36-3.31) | 0.88 | 0.42 (0.10-1.76) | 0.24 | 0.78 (0.14-4.36) | 0.78 | 0.59 (0.18-1.88) | 0.37 |
| Definite/probable ST | 3 (1.3) | 11 (1.2) | 1.13 (0.31-4.09) | 0.85 | 0.47 (0.13-1.72) | 0.25 | 1.33 (0.22-7.98) | 0.75 | 0.95 (0.25-3.56) | 0.94 |

Data are presented as mean (SD, or n (%).
^a^Included (cardiac death, TV-MI, or ID-TLR). †Included (all-cause death, any recurrent MI, or any revascularization).
^b^The confounding factors considered in the adjusted hazard ratio are age, sex, BMI, clinical presentation, hypertension, diabetes mellitus, dyslipidemia, current smoker, prior MI, prior revascularization, prior CVA, LVEF, eGFR, CK-MB, LDL-cholesterol, CRP, DAPT, aspirin, P2Y12 inhibitor, ACEi or ARB, beta-blocker, statin, multi-vessel disease, culprit vessel, B2/C lesion, trans-radial approach, glycoprotein IIb/IIIa inhibitor, thrombus aspiration, thrombus type, successful PCI, multiple stent implantation, stent length ≥ 60mm, IABP, ECMO, in-hospital CPR and length of hospital stays.

Abbreviations: ID-TLR, ischemic-driven target lesion revascularization; IVUS, intravascular ultrasound; MACE, major adverse cardiac events; MI, myocardial infarction; ST, stent thrombosis; TLF, target lesion failure; TV-MI, target vessel myocardial infarction.

**Supplemental Table S4. One-year clinical outcome in patients with non-ST-segment elevation myocardial infarction**

|  | IVUS-guided (n=259) | Angiography-guided (n=885) | Unadjusted | | Multivariable-Adjusted^b^ | | PS-Matched | | IPTW-Adjusted | |
| --- | --- | --- | --- | --- | --- | --- | --- | --- | --- | --- |
|  |  |  | **Hazard ratio (95% CI)** | ***p* value** | **Hazard ratio (95% CI)** | ***p* value** | **Hazard ratio (95% CI)** | ***p* value** | **Hazard ratio (95% CI)** | ***p* value** |
| **Primary outcome** |  |  |  |  |  |  |  |  |  |  |
| Target lesion failure^a^ | 9 (12.9) | 51 (24.3) | 0.48 (0.23-0.97) | 0.04 | 0.53 (0.19-1.48) | 0.23 | 1.24 (0.35-4.43) | 0.74 | 0.66 (0.28-1.53) | 0.33 |
| **Secondary outcome** |  |  |  |  |  |  |  |  |  |  |
| MACE† | 15 (21.4) | 74 (35.2) | 0.54 (0.31-0.95) | 0.03 | 0.51 (0.24-1.08) | 0.08 | 1.42 (0.56-3.64) | 0.46 | 0.62 (0.32-1.20) | 0.15 |
| All-cause death | 9 (12.9) | 60 (28.6) | 0.40 (0.20-0.81) | 0.01 | 0.37 (0.13-1.07) | 0.07 | 0.86 (0.29-2.55) | 0.79 | 0.59 (0.26-1.33) | 0.20 |
| Cardiac death | 7 (10.0) | 45 (21.4) | 0.42 (0.19-0.94) | 0.04 | 0.59 (0.16-2.14) | 0.42 | 1.60 (0.43-5.96) | 0.48 | 0.71 (0.29-1.75) | 0.45 |
| Any MI | 4 (5.7) | 8 (3.8) | 1.31 (0.39-4.35) | 0.66 | 0.20 (0.05-0.76) | 0.02 | 2.00 (0.13-31.98) | 0.62 | 0.68 (0.19-2.47) | 0.55 |
| TV-MI | 2 (2.9) | 2 (1.0) | 2.52 (0.35-17.86) | 0.36 | NA | NA | NA | NA | 0.82 (0.11-6.35) | 0.85 |
| Any revascularization | 4 (5.7) | 14 (6.7) | 0.72 (0.24-2.20) | 0.57 | 0.68 (0.22-2.14) | 0.51 | NA | NA | 0.50 (0.15-1.67) | 0.26 |
| ID-TLR | 1 (1.4) | 5 (2.4) | 0.51 (0.06-4.35) | 0.54 | NA | NA | NA | NA | 0.27 (0.03-2.38) | 0.24 |
| Definite/probable ST | 2 (2.9) | 2 (1.0) | 2.85 (0.40-20.22) | 0.30 | NA | NA | 2.00 (0.13-31.98) | 0.62 | 1.71 (0.23-12.54) | 0.60 |

Data are presented as mean (SD, or n (%).
^a^Included (cardiac death, TV-MI, or ID-TLR). †Included (all-cause death, any recurrent MI, or any revascularization).
^b^The confounding factors considered in the adjusted hazard ratio are age, sex, BMI, clinical presentation, hypertension, diabetes mellitus, dyslipidemia, current smoker, prior MI, prior revascularization, prior CVA, LVEF, eGFR, CK-MB, LDL-cholesterol, CRP, DAPT, aspirin, P2Y12 inhibitor, ACEi or ARB, beta-blocker, statin, multi-vessel disease, culprit vessel, B2/C lesion, trans-radial approach, glycoprotein IIb/IIIa inhibitor, thrombus aspiration, thrombus type, successful PCI, multiple stent implantation, stent length ≥ 60mm, IABP, ECMO, in-hospital CPR and length of hospital stays.

Abbreviations: ID-TLR, ischemic-driven target lesion revascularization; IVUS, intravascular ultrasound; MACE, major adverse cardiac events; MI, myocardial infarction; ST, stent thrombosis; TLF, target lesion failure; TV-MI, target vessel myocardial infarction.

**Supplemental Table S5. Clinical outcome at 30 days**

|  | **IVUS-guided (n=294)** | **Angio-guided (n=1,124)** | **Unadjusted** | | **Multivariable-Adjusted^b^** | | **PS-Matched** | | **IPTW-Adjusted** | |
| --- | --- | --- | --- | --- | --- | --- | --- | --- | --- | --- |
|  |  |  | **Hazard ratio (95% CI)** | ***p* value** | **Hazard ratio (95% CI)** | ***p* value** | **Hazard ratio (95% CI)** | ***p* value** | **Hazard ratio (95% CI)** | ***p* value** |
| **Primary outcome** |  |  |  |  |  |  |  |  |  |  |
| Target lesion failure^a^ | 36 (12.2) | 218 (19.4) | 0.59 (0.42-0.85) | <0.01 | 0.88 (0.60-1.28) | 0.50 | 0.98 (0.63-1.53) | 0.94 | 0.97 (0.65-1.46) | 0.89 |
| **Secondary outcome** |  |  |  |  |  |  |  |  |  |  |
| MACE† | 41 (13.9) | 247 (22.0) | 0.61 (0.44-0.84) | <0.01 | 0.94 (0.66-1.34) | 0.73 | 0.88 (0.58-1.34) | 0.55 | 0.94 (0.64-1.38) | 0.74 |
| All-cause death | 35 (11.9) | 239 (21.3) | 0.53 (0.37-0.76) | <0.01 | 0.65 (0.43-0.97) | 0.03 | 0.99 (0.61-1.59) | 0.95 | 0.87 (0.58-1.30) | 0.50 |
| Cardiac death | 35 (11.9) | 217 (19.3) | 0.58 (0.41-0.83) | <0.01 | 0.86 (0.59-1.26) | 0.45 | 0.98 (0.63-1.53) | 0.94 | 0.97 (0.64-1.46) | 0.88 |
| Any MI | 8 (2.7) | 10 (0.9) | 3.06 (1.21-7.75) | 0.02 | 4.44 (1.33-14.75) | 0.02 | 7.12 (0.79-64.38) | 0.08 | 2.83 (1.05-7.65) | 0.04 |
| TV-MI | 1 (0.3) | 1 (0.1) | 3.44 (0.22-55.07) | 0.38 | 2.88 (0.18-46.03) | 0.45 | NA | NA | 2.19 (0.14-35.02) | 0.58 |
| Any revascularization | 3 (1.0) | 3 (0.3) | 3.45 (0.70-17.11) | 0.13 | NA | NA | 2.00 (0.28-14.20) | 0.49 | 3.63 (0.61-21.71) | 0.16 |
| ID-TLR | 2 (0.7) | 1 (0.1) | 6.90 (0.63-76.09) | 0.11 | NA | NA | NA | NA | 4.09 (0.37-45.30) | 0.25 |
| Definite/probable ST | 5 (1.7) | 10 (0.9) | 1.73 (0.59-5.06) | 0.32 | 0.63 (0.22-1.85) | 0.40 | 2.30 (0.38-14.12) | 0.37 | 1.70 (0.55-5.24) | 0.35 |

Data are presented as mean (SD) or n (%).
^a^Included (cardiac death, TV-MI, or ID-TLR). †Included (all-cause death, any recurrent MI, or any revascularization).
^b^The confounding factors considered in the adjusted hazard ratio are age, sex, BMI, clinical presentation, hypertension, diabetes mellitus, dyslipidemia, current smoker, prior MI, prior revascularization, prior CVA, LVEF, eGFR, CK-MB, LDL-cholesterol, CRP, DAPT, aspirin, P2Y12 inhibitor, ACEi or ARB, beta-blocker, statin, multi-vessel disease, culprit vessel, B2/C lesion, trans-radial approach, glycoprotein IIb/IIIa inhibitor, thrombus aspiration, thrombus type, successful PCI, multiple stent implantation, stent length ≥ 60mm, IABP, ECMO, in-hospital CPR and length of hospital stays.
Abbreviations: ID-TLR, ischemic-driven target lesion revascularization; IVUS, intravascular ultrasound; MACE, major adverse cardiac events; MI, myocardial infarction; ST, stent thrombosis; TLF, target lesion failure; TV-MI, target vessel myocardial infarction.

**Supplemental Table S6. Clinical outcome between 30 days and 1 year (landmark analysis)**

|  | IVUS-guided (n=259) | Angiography-guided (n=885) | Unadjusted | | Multivariable-Adjusted^b^ | | PS-Matched | | IPTW-Adjusted | |
| --- | --- | --- | --- | --- | --- | --- | --- | --- | --- | --- |
|  |  |  | **Hazard ratio (95% CI)** | ***p* value** | **Hazard ratio (95% CI)** | ***p* value** | **Hazard ratio (95% CI)** | ***p* value** | **Hazard ratio (95% CI)** | ***p* value** |
| **Primary outcome** |  |  |  |  |  |  |  |  |  |  |
| Target lesion failure^a^ | 12 (4.7) | 45 (5.1) | 0.94 (0.49-1.77) | 0.84 | 0.59 (0.28-1.22) | 0.15 | 0.47 (0.17-1.27) | 0.14 | 0.77 (0.38-1.53) | 0.45 |
| **Secondary outcome** |  |  |  |  |  |  |  |  |  |  |
| MACE† | 23 (9.1) | 102 (11.6) | 0.81 (0.51-1.27) | 0.36 | 0.66 (0.40-1.07) | 0.09 | 0.70 (0.37-1.31) | 0.27 | 0.72 (0.44-1.19) | 0.20 |
| All-cause death | 14 (5.4) | 47 (5.3) | 1.05 (0.58-1.91) | 0.87 | 0.81 (0.41-1.63) | 0.56 | 0.76 (0.33-1.79) | 0.53 | 0.96 (0.50-1.86) | 0.90 |
| Cardiac death | 8 (3.1) | 26 (2.9) | 1.09 (0.49-2.40) | 0.84 | 0.60 (0.21-1.70) | 0.33 | 0.61 (0.19-1.97) | 0.41 | 1.09 (0.47-2.55) | 0.84 |
| Any MI | 2 (0.8) | 10 (1.1) | 0.76 (0.16-3.46) | 0.72 | NA | NA | NA | NA | 0.33 (0.07-1.61) | 0.17 |
| TV-MI | 2 (0.8) | 3 (0.3) | 2.31 (0.39-13.83) | 0.36 | NA | NA | NA | NA | 0.93 (0.14-6.18) | 0.94 |
| Any revascularization | 11 (4.3) | 59 (6.6) | 0.67 (0.35-1.28) | 0.23 | 0.54 (0.27-1.09) | 0.08 | 0.55 (0.22-1.39) | 0.21 | 0.57 (0.29-1.13) | 0.11 |
| ID-TLR | 3 (1.2) | 18 (2.0) | 0.58 (0.17-1.98) | 0.39 | 0.32 (0.08-1.26) | 0.10 | 0.25 (0.03-2.00) | 0.19 | 0.37 (0.11-1.30) | 0.12 |
| Definite/probable ST | 0 (0.0) | 3 (0.3) | NA | NA | NA | NA | NA | NA | NA | NA |

Data are presented as mean (SD, or n (%).
^a^Included (cardiac death, TV-MI, or ID-TLR). †Included (all-cause death, any recurrent MI, or any revascularization).
^b^The confounding factors considered in the adjusted hazard ratio are age, sex, BMI, clinical presentation, hypertension, diabetes mellitus, dyslipidemia, current smoker, prior MI, prior revascularization, prior CVA, LVEF, eGFR, CK-MB, LDL-cholesterol, CRP, DAPT, aspirin, P2Y12 inhibitor, ACEi or ARB, beta-blocker, statin, multi-vessel disease, culprit vessel, B2/C lesion, trans-radial approach, glycoprotein IIb/IIIa inhibitor, thrombus aspiration, thrombus type, successful PCI, multiple stent implantation, stent length ≥ 60mm, IABP, ECMO, in-hospital CPR and length of hospital stays.

Abbreviations: ID-TLR, ischemic-driven target lesion revascularization; IVUS, intravascular ultrasound; MACE, major adverse cardiac events; MI, myocardial infarction; ST, stent thrombosis; TLF, target lesion failure; TV-MI, target vessel myocardial infarction.
